# Myocardial Expression of PPAR*γ* and Exercise Capacity in Patients after Coronary Artery Bypass Surgery

**DOI:** 10.1155/2017/1924907

**Published:** 2017-09-28

**Authors:** Izabela Wojtkowska, Tomasz A. Bonda, Jadwiga Wolszakiewicz, Jerzy Osak, Andrzej Tysarowski, Katarzyna Seliga, Janusz A. Siedlecki, Maria M. Winnicka, Ryszard Piotrowicz, Janina Stępińska

**Affiliations:** ^1^Institute of Cardiology, Intensive Cardiac Therapy Clinic, Alpejska St. 42, 04-628 Warsaw, Poland; ^2^Department of General and Experimental Pathology, Medical University of Bialystok, Mickiewicza St. 2c, 15-222 Bialystok, Poland; ^3^Institute of Cardiology, Department of Cardiac Rehabilitation and Noninvasive Electrocardiology, Alpejska St. 42, 04-628 Warsaw, Poland; ^4^Institute of Oncology, Department of Molecular and Translational Oncology, Wawelska St. 15B, 02-034 Warsaw, Poland

## Abstract

Activation of PPARs may be involved in the development of heart failure (HF). We evaluated the relationship between expression of PPAR*γ* in the myocardium during coronary artery bypass grafting (CABG) and exercise tolerance initially and during follow-up. 6-minute walking test was performed before CABG, after 1, 12, 24 months. Patients were divided into two groups (HF and non-HF) based on left ventricular ejection fraction and plasma proBNP level. After CABG, 67% of patients developed HF. The mean distance 1 month after CABG in HF was 397 ± 85 m versus 420 ± 93 m in non-HF. PPAR*γ* mRNA expression was similar in both HF and non-HF groups. 6MWT distance 1 month after CABG was inversely correlated with PPAR*γ* level only in HF group. Higher PPAR*γ* expression was related to smaller LVEF change between 1 month and 1 year (*R* = 0.18, *p* < 0.05), especially in patients with HF. Higher initial levels of IL-6 in HF patients were correlated with longer distance in 6MWT one month after surgery and lower PPAR*γ* expression. PPAR*γ* expression is not related to LVEF before CABG and higher PPAR*γ* expression in the myocardium of patients who are developing HF following CABG may have some protecting effect.

## 1. Introduction

Heart failure (HF) is characterized by reduced reserve of the cardiac output. Impaired functional capacity in patients with heart failure is common and results from inability to achieve sufficient oxygen and nutrients delivery and altered washout of metabolites from working muscles.

Decreased stroke volume, altered chronotropic reaction, insufficient increase of myocardial contractility, and altered left ventricular-aortic coupling constitute the major central cardiovascular abnormalities, while decreased capillary density, endothelial dysfunction, and lowered oxygen extraction create the peripheral basic pathomechanisms leading to insufficient oxygenation of skeletal muscles [1, 2]. In addition, chronic HF is related to dysfunctional metabolism of the skeletal muscles, changes in fibre composition, and progressive muscular atrophy [3, 4]. Overproduction of proinflammatory cytokines like TNF-*α* or IL-6, which is characteristic for chronic HF, may be responsible for altered muscular structure and function [5, 6]. These cytokines have an influence on normal physiology of the skeletal muscle cells but also affect proper function of endothelium by promoting generation of free oxygen radicals and decreasing availability of nitric oxide that altogether are responsible for insufficient vasorelaxatory function of the blood vessels [7]. The unfavorable effects of chronic inflammation in heart failure may be opposed by different endogenous mechanisms. One of these mechanisms may be related to the function of peroxisome proliferator-activated receptor gamma (PPAR*γ*), which gained attention in recent decade mainly because of its metabolism-improving activities. Thiazolidinediones, the pharmacological activators of PPAR*γ*, are used in patients with diabetes to decrease insulin resistance and improve glycemic control. Some positive effects of this therapy were also noted in relation to the cardiovascular system. Thiazolidinediones improved function of the endothelium, enhanced fatty acid oxidation in the cardiac muscle, decreased myocardial fibrosis, and diminished the risk of myocardial infarction and stroke [8]. Numerous experimental studies using rodents showed protective activity of PPAR*γ* agonists on cardiac function [9–12]. However the significance of PPAR*γ* activation is not univocal, as pioglitazone failed to provide any protective effect on the myocardium after ischemia-reperfusion in pigs [13] and rosiglitazone failed to prevent cardiac remodeling and caused increased mortality after acute infarction in rats [14]. Moreover, a meta-analysis of clinical studies showed increased risk for developing heart failure in patients with diabetes treated with PPAR*γ* agonist pioglitazone [15]. The mechanisms leading to development of heart failure in patients treated with pioglitazone remain vague. These data were further blurred by improvement of aerobic capacity and skeletal muscle energy metabolism in patients with metabolic syndrome treated with pioglitazone [16].

In the present study we aimed to evaluate the relationship between expression of PPAR*γ* in the myocardium, plasma levels of IL-6, and exercise tolerance in patients with ischemic heart disease undergoing coronary artery bypass grafting (CABG) before the operation and during the follow-up.

## 2. Methods

Patients with angiographically confirmed multivessel coronary artery disease, qualified to the CABG, were recruited to the study. Only subjects with normal blood levels of NT-proBNP and preserved cardiac function in resting echocardiographic examination and without clinical manifestations of heart failure or diabetes mellitus were included.

The clinical examination, biochemical tests, resting echocardiography, and six-minute walk test (6MWT) were performed before CABG and at 3-time points during the follow-up: one, twelve, and twenty-four months after the operation.

6MWT is a submaximal exercise test for evaluation of physical functional capacity measured in walked distance. The methodology of the examination was in agreement with the published guidelines [17]. Briefly, before the test patients were informed about the procedure and were allowed to rest in a sitting position for 10 minutes. Then they were asked to walk as fast and long as possible on a 50-meter walkway. Patients were allowed to stop and rest or reduce their walking speed if they felt fatigue. The dyspnea was estimated using the Borg scale. Samples of the left ventricular myocardium were harvested during the CABG procedure, and tissue fragments were placed in the “RNA later” solution (Qiagen) immediately after surgery and stored until RNA isolation. Expression of PPAR*γ* mRNA was determined in these samples by means of quantitative real-time PCR using TaqMan probes as previously described [18].

Blood samples were drawn initially and during each follow-up step. Serum concentrations of IL6 were measured using solid phase sandwich enzyme-linked immunosorbent assay kits (HS600B, R&D Systems) according to the manufacturer's guidelines.

Patients were examined during the follow-up and the measures of heart failure development were sought. During follow-up all patients were divided into two groups: with heart failure (HF) and without heart failure (non-HF). The criteria for the diagnosis of heart failure were left ventricular ejection fraction (LVEF) < 40% or NT-proBNP > 400 pg/mL. Presence of any of the abovementioned values during the follow-up was considered a marker of heart failure.

### 2.1. Ethics Statement

The procedures followed in the study were conducted ethically according to the principles of the World Medical Association Declaration of Helsinki and ethical standards in sport and exercise science research. All procedures were approved by the Ethics Committee of the Regional Medical Chamber in Warsaw [IK NP-0021/13/998/2007]. Informed consent was obtained from all participants.

### 2.2. Statistics

Data are presented as mean ± SD for quantitative variables or percent of study group for qualitative variables. Specific parameters of both groups (group with and without heart failure at baseline) and change in parameter values during follow-up were compared using chi square test and ANOVA with post hoc analysis. Correlations between variables were tested using Pearson's method. A value of *p* < 0.05 was considered statistically significant. Analysis was performed using Statistica 9.0PL.

## 3. Results

**Figure 1:**
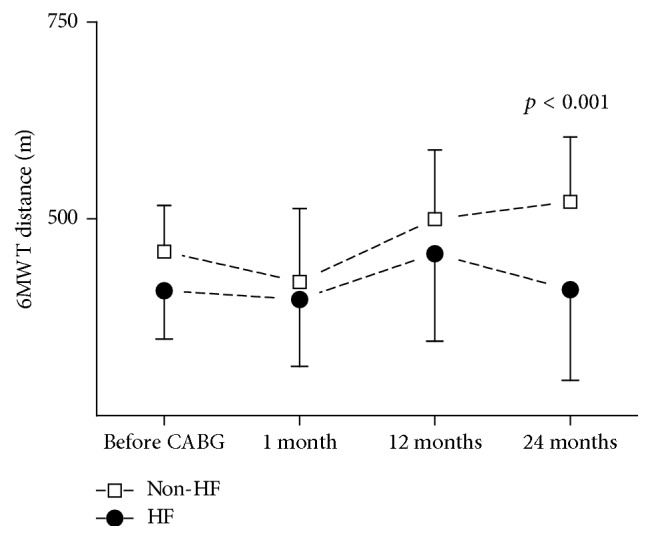
Changes of distance in 6-minute waking test in patients with (HF) and without heart failure (non-HF) during the follow-up. The improvement of the distance 24 months after CABG was observed only in non-HF group, and the distance was significantly longer as compared to the HF patients.

**Figure 2:**
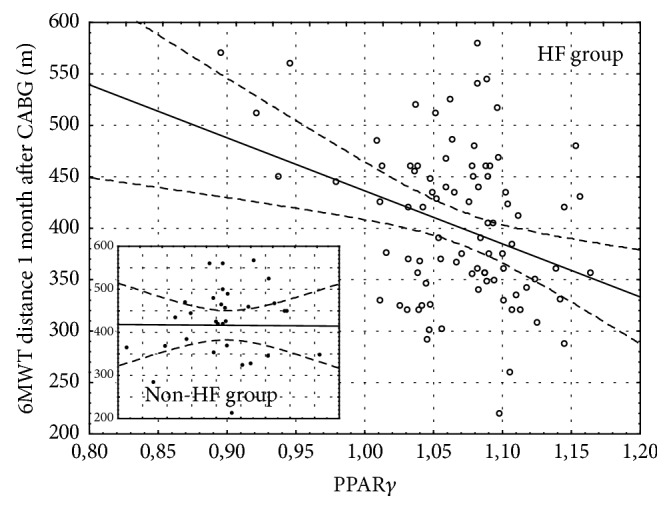
6MWT distance 1 month after CABG was negatively correlated with PPAR*γ* only in patients with HF during follow-up (*R* = −0.24; *p* < 0.05).

**Figure 3:**
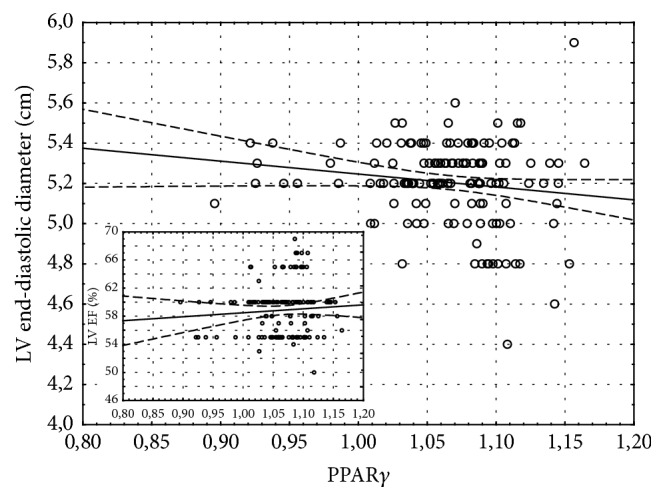
There were no significant correlations between PPAR*γ* and either left ventricular end-diastolic dimension (*R* = −0.11, *p* = NS, the main graph) or left ventricular ejection fraction (LVEF; *R* = 0.05, *p* = NS, small graph) after CABG.

**Figure 4:**
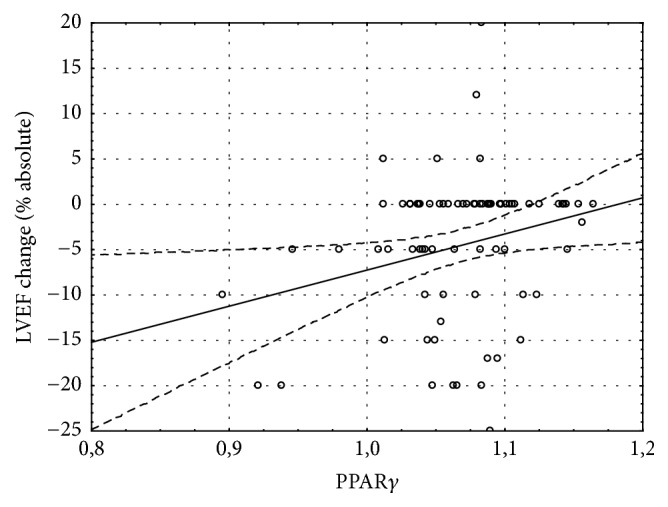
LVEF change between 1- and 12-month follow-up was significantly correlated with myocardial PPAR*γ* in patients, in whom heart failure was diagnosed (*R* = 0.25, *p* < 0.05).

**Figure 5:**
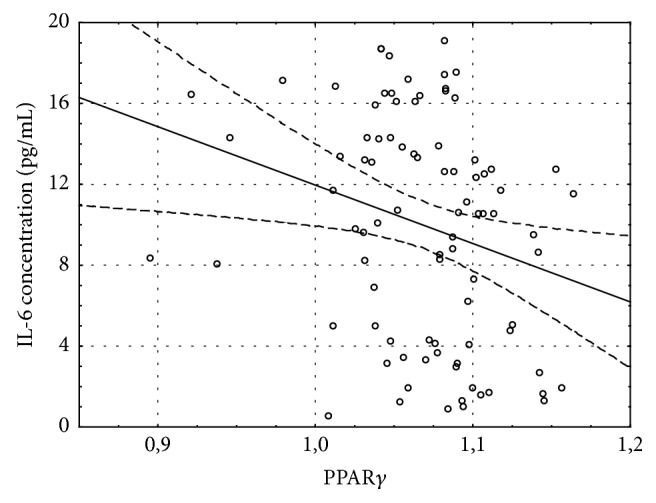
Significant negative correlation between serum IL6 level before CABG and expression of PPAR*γ* (*R* = −0.31, *p* < 0.05) was observed only in patients in whom later in the follow-up heart failure was diagnosed.

157 patients were qualified to the study. All patients did not have heart failure before CABG. After 1 month of CABG, 67% of patients developed heart failure. During 2-year follow-up the number of patients has been reduced in 1 year to 124 and in 2 years to 86 because of the loss of connection. In the HF group one patient died because of myocardial infarction in 1 year after CABG. The baseline characteristics of the study group were presented in the previous paper [18]. One month after CABG 106 patients (67%) were diagnosed with heart failure based on NT-proBNP exceeding 400 pg/mL or LVEF < 40%. Mean NT-proBNP concentration in this group was 675.2 ± 134.7 pg/mL and increased NT-proBNP was the most frequent indicator of HF. Only 13 subjects out of 106 had LVEF < 40%. The initial distance in 6MWT was 439 ± 73 m (408 ± 61 m in HF group and 458 ± 59 m in non-HF group). Patients developing HF during the follow-up had insignificantly lower exercise capacity 1 month after CABG than patients without HF. The mean 6MWT distance in HF group was 397 ± 85 m versus 420 ± 93 m in patients without HF. The distance improved significantly during the follow-up only in patients without HF (*p* = 0.002) and 24 months after CABG it was significantly longer than in HF group (410 ± 134 m in HF group versus 522 ± 82 m in non-HF group, *p* < 0.001, Figure 1). Rate of perceived exertion scale was 6–12 for majority of patients. The parameters of 6MWT in both HF and non-HF groups are presented in Table 1. PPAR*γ* gene expression in left ventricular myocardium taken during CABG was at the same level in patients included in HF and non-HF group during follow-up (1.069 ± 0.049 and 1.069 ± 0.034, resp.). However the distance of 6MWT one month after CABG was inversely correlated with myocardial PPAR*γ* level (*R* = −0.24, *p* < 0.05) only in patients developing HF during the follow-up and was not present in those without HF (Figure 2). Although the expression of PPAR*γ* gene in the myocardium was not correlated with left ventricular ejection fraction (LVEF) before or after CABG (Figure 3), higher PPAR*γ* level was related to smaller attenuation of LVEF between 1-month and 1-year observations (*R* = 0.18, *p* < 0.05). The relation described was more pronounced in those patients, who developed HF during the follow-up (*R* = 0.25, *p* < 0.01) and was not significant in patients without HF (Figure 4). The concentration of IL-6 was highest before CABG (7.2 ± 5.5 pg/mL) and was significantly declining during the follow-up to reach level of 2.4 ± 2.1 pg/mL after one year and remained at that level (*p* < 0.0001 for the trend). Before CABG there was negative correlation between concentration of IL-6 in plasma and PPAR*γ* expression (*R* = −0.31, *p* < 0.05, Figure 5) and positive correlation between IL-6 and NT-proBNP (*R* = 0.35, *p* < 0.001). In patients who developed heart failure higher initial levels of IL-6 were correlated with longer distance in 6MWT one month after surgery (*R* = 0.47, *p* < 0.001).

## 4. Discussion

The six-minute walk test (6MWT) is a submaximal exercise test for evaluating physical functional capacity. Fiorina et al. suggest that 6MWT is feasible and well tolerated in adult and older patients shortly after uncomplicated cardiac surgery and provides reference values for distance walked after cardiac surgery [19]. In our observations patients diagnosed with heart failure after CABG had a shorter distance in 6MWT, than patients without heart failure. In the HF group there was no significant improvement of the distance in 6MWT, while in patients without HF the distance increased significantly. Differences in exercise capacity can be attributed to the altered cardiac function; however there were no correlations between distance in 6MWT and LVEF or LV diastolic dimension. It should be emphasized that the abovementioned two parameters are related to the systolic function of the heart and poorly related to its diastolic performance.

Literature describing potential links between myocardial PPAR*γ* expression and cardiac function or exercise capacity after CABG is very scant. In addition existing experimental data present contrasting results about the influence of PPAR*γ* on cardiac function. On one hand, overexpression of PPAR*γ* in transgenic mice was reported to evoke accumulation of lipids and glycogen and distortion of mitochondrial architecture leading to dilated cardiomyopathy [20]. On the other hand, activation of PPAR*γ* with its agonist reduced lipotoxicity and improved cardiac function [21]. Expression of PPARs and their coactivators is diminished in cardiomyopathy and heart failure [22]. Cernecka et al. reported downregulation of PPAR*γ* in the myocardium of anthracycline-induced cardiomyopathy in rodents, and treatment with an angiotensin converting enzyme inhibitor restored normal level of PPAR*γ* but was not sufficient to restore normal cardiac function [23].

Our data showed no clear relation between PPAR*γ* expression at the time of CABG and left ventricular size or ejection fraction at any time point. The level of PPAR*γ* in the myocardium was not able to predict development of heart failure after CABG, as was shown in our previous work [18]. However, in the group of patients, in whom heart failure develops during the follow-up, higher myocardial PPAR*γ* level seems to preserve the systolic function of the left ventricle. On the other hand, also only in patients developing HF, PPAR*γ* expression was inversely correlated with exercise capacity during the follow-up. It should be emphasized that left ventricular systolic function, if not significantly depressed, is not related to the exercise tolerance, but abnormalities of its diastolic function are independently associated with exercise capacity [24]. In our study however, NT-proBNP, which can be considered a marker of increased myocardial strain during systole and during diastole, was not related to PPAR*γ* in the myocardium at all. Possible protective effect of PPAR*γ* on the cardiac function may be related to suppression of the excessive inflammatory process and inhibition of apoptosis [25, 26]. Persistent inflammation, with chronically elevated concentrations of proinflammatory cytokines like TNF-*α*, ET-1, and IL-6, plays a pathogenic role in heart failure [27, 28]. In patients with heart failure, inflammation has been associated with worse functional capacity [29, 30] and concomitant cytokines and angiotensin II overproduction was shown to promote skeletal muscle atrophy in animals' models [31] that resemble changes seen in chronic heart failure. Without heart failure however, IL-6 seems to exert protective role on the skeletal muscles, stimulates hypertrophy and myogenesis, and regulates the energy metabolism [31]. We found negative correlation between myocardial PPAR*γ* expression and IL-6 level only before CABG, when none of patients had features of heart failure. A subgroup of patients who developed HF and had higher initial levels of IL-6 had also better exercise capacity at one-month follow-up, which may reflect the protective role of IL-6 on skeletal muscle physiology, which is more important for skeletal muscle function and exercise capacity than weak PPAR*γ*-related phenomena affecting cardiac muscle.

## 5. Conclusions

Higher levels of PPAR*γ* in myocardium of patients who developed HF after CABG were correlated with smaller attenuation of LVEF, reduced plasma level of IL-6, and worsening of exercise tolerance.

These results indicate that PPAR*γ* expression in the myocardium was not related to left ventricular systolic function before CABG. However higher levels of PPAR*γ* gene transcript in the myocardium of patients who develop heart failure following CABG may have some protecting effect on cardiac contractility, which seem not to be directly related to exercise capacity.

## Figures and Tables

**Table 1:** Temporal changes of parameters related to six-minute walk test (6MWT) in patients with heart failure (HF) and without heart failure (non-HF).

	Before CABG *n* = 157	HF					Non-HF				
		Before CABG *n* = 106	After CABG				Before CABG *n* = 33	After CABG			
			1 month *n* = 106	1 year *n* = 91	2 years *n* = 54	*p*		1 month *n* = 33	1 year *n* = 33	2 years *n* = 32	*p*
6MWT distance (±SD) [m]	439 (±73)	408 (±61)	397 (±85)	456 (±110)	410 (±134)	NS	458 (±59)	420 (± 93)	499 (± 87)	522 (± 82)	0.002

Rate of perceived exertion scale (Borg)											
6–12	90%	75%		80%	75%	NS	80%		85%	90%	NS
12–16	10%	20%		15%	20%		20%		15%	10%	
17–20	0%	5%		5%	5%		0%		0%	0%	

Respiratory rate/min.											
<14	90%	75%		80%	75%	NS	80%		85%	90%	NS
<20	10%	20%		15%	20%		20%		15%	10%	
<25	0%	5%		5%	5%		0%		0%	0%	

HR/min.											
<100	90%	75%		80%	75%	NS	80%		85%	90%	NS
<120	10%	20%		15%	20%		20%		15%	10%	
<160	0%	5%		5%	5%		0%		0%	0%	

## Additional Points

*Study Limitations. *Significant number of patients was lost during follow-up: 12% 1 month after CABG, 18% after one year, and 36% after two years. The significance of the PPAR*γ* expression may not be directly translated into its activity and biological role. The correlations presented in the work do not imply the causality and may be accidental; however it is not possible to evaluate them in the clinical observational study.
